# Selection of sampling points for saturation recovery based myocardial T_1 _mapping

**DOI:** 10.1186/1532-429X-16-S1-W32

**Published:** 2014-01-16

**Authors:** Mehmet Akcakaya, Sebastian Weingartner, Warren J Manning, Reza Nezafat

**Affiliations:** 1 Medicine, Beth Israel Deaconess Medical Center, Harvard Medical School, Boston, Massachusetts, USA; 2 Computer Assisted Clinical Medicine, University Medical Center Mannheim, Heidelberg University, Mannheim, Germany; 3 Radiology, Beth Israel Deaconess Medical Center, Harvard Medical School, Boston, Massachusetts, USA

## Background

Quantitative myocardial T_1 _mapping allows assessment of focal and diffuse fibrosis in the myocardium, by sampling the T_1 _relaxation curve using inversion [1] or saturation recovery (SR) preparation [2] or a combination of both [3], followed by the acquisition of multiple images with different contrasts, which are subsequently fitted to a parametric equation pixel-wise to yield the T_1 _maps. In myocardial T_1 _mapping, there is a degree of freedom in selecting which points on the relaxation curve are sampled. However, this topic has not been studied. In this study, we sought to develop an estimation theoretic framework for optimal selection of sampling points and characterized the variance of the corresponding T_1 _estimator for sampling of the SR curve.

## Methods

Based on the signal model, y_k _= a (1-b exp(-x_k_/T_1_))+n_k_, and the least squares model, we derived the Fisher information matrix [4]. This was used to derive the Bayesian Cramer-Rao bound [4] for the variance of the T_1 _estimator for T_1 _values of interest between 950 and 1250 ms (~pre-contrast myocardium). The bound was evaluated for the SASHA sequence [2] which allows sampling within a heart-beat between T_min _and T_max _with one point at full magnetization recovery (x_k _= ∞), and minimized over the choice of sampling points {x_k_} yielding the proposed point selection. Phantom imaging of NiCl_2 _doped agarose vials was performed to compare the proposed point selection with a uniform distribution of sampling points between T_min _and T_max _[3] using an SSFP sequence with body-coil (NSA = 5) for 11 sampling points. Standard deviation (std) of T_1 _values within the vials was used as a surrogate for the variance of the estimator. Imaging was also performed on 5 healthy adult subjects (4 women, 23.4 ± 3.3 years) with a 32-channel cardiac-coil to verify the gains predicted by the theory. Both proposed and uniform point selection acquisitions were repeated 5 times per subject to average out the effects of noise. ROIs were drawn in the myocardium and the blood. Both the T_1 _estimate (average T_1 _values in the ROI) and the std of the estimator (std of T_1 _values in the ROI) are reported as mean ± std across 5 scans.

## Results

**Figure 1:**
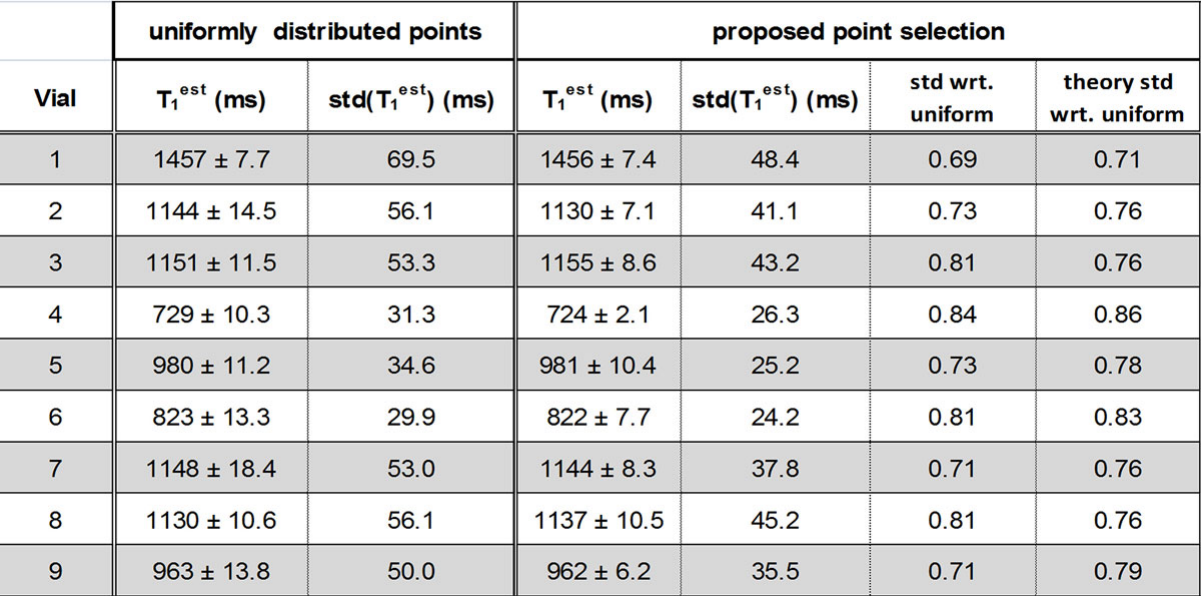
**Results of the phantom imaging over vials with T_1 _values > 700 ms using the proposed and uniform sampling strategies, where each acquisition was repeated 5 times**. The ratio of the standard deviation of the T_1 _estimator for each proposed sampling strategy and that of the uniform sampling strategy is reported as "standard deviation (std) with respect to (wrt) uniform." There is a gain in using the proposed point selection strategy, which is significantly different than 1 (P < 0.001). The values match those predicted by theory (P = 0.23).

**Figure 2:**
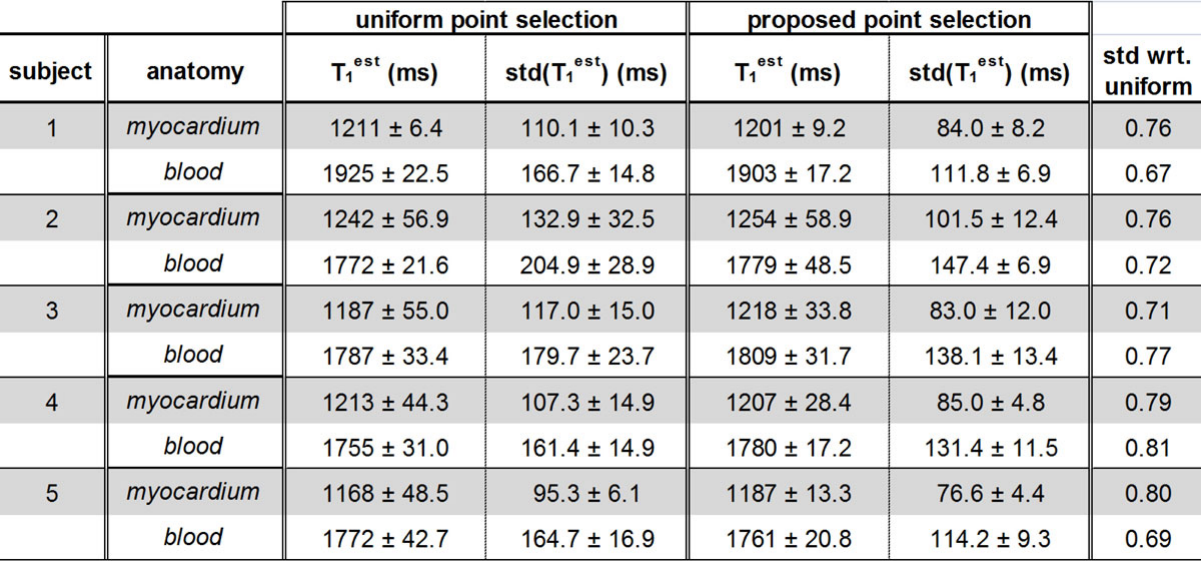
**Results of in-vivo imaging for five healthy subjects using the proposed and uniform sampling strategies, where each acquisition was repeated 5 times**. T_1_^est ^is reported as the mean ± std of the average T_1 _values in the ROI across 5 scans, as a surrogate for accuracy and inter-scan reproducibility. The std(T_1_^est^) is reported as the mean ± std of the std of the T_1 _values in the ROI across 5 scans, as a surrogate for the precision within the scan. Std wrt. uniform is the ratio of the mean values of std(T_1_^est^) using the proposed and uniform point selection, as a surrogate for the percentage gain in precision. The standard deviation of the T_1 _estimator in the myocardium and blood was reduced by 23.6% and 26.8% respectively using the proposed approach.

The point selection yielded a tri-modal distribution of points: 4 at T_min_, 6 at T_max_, 1 at ∞, with a theoretical gain in std of 24% compared to uniform selection. Figure 1 shows the results of phantom imaging for T_1 _values > 700 ms, indicating a good match between theory and experiment. Figure 2 depicts the measurements from the in-vivo data, averaged over five scans. Overall, there was a 23.6% and 26.8% reduction in the std of the T_1 _maps in the myocardium and blood respectively using the proposed approach.

## Conclusions

The proposed framework allows for choosing the location of points on the T_1 _relaxation curve to achieve higher levels of precision without increasing the scan time.

## Funding

NIH:K99HL111410-01; R01EB008743-01A2.
